# Network-based prediction and knowledge mining of disease genes

**DOI:** 10.1186/1755-8794-8-S2-S9

**Published:** 2015-05-29

**Authors:** Matthew B Carson, Hui Lu

**Affiliations:** 1 Department of Bioengineering/Bioinformatics, University of Illinois at Chicago, 835 S. Wolcott, Chicago, IL 60612, USA; 2 Division of Health and Biomedical Informatics, Department of Preventive Medicine, Northwestern University Feinberg School of Medicine, 750 N. Lake Shore Drive, Chicago, IL 60611, USA; 3 Center for Healthcare Studies, Institute for Public Health and Medicine, Northwestern University Feinberg School of Medicine, 633 N. Saint Clair, Chicago, IL 60611, USA; 4 Shanghai Institute of Medical Genetics, Shanghai Children's Hospital, Shanghai Jiaotong University, Shanghai 200040, China; 5 Collaborative Innovation Center for Biotherapy, West China Hospital, Sichuan University, Chengdu, China

**Keywords:** Protein interaction, networks, Disease Ontology, ADTree

## Abstract

**Background:**

In recent years, high-throughput protein interaction identification methods have generated a large amount of data. When combined with the results from other in vivo and in vitro experiments, a complex set of relationships between biological molecules emerges. The growing popularity of network analysis and data mining has allowed researchers to recognize indirect connections between these molecules. Due to the interdependent nature of network entities, evaluating proteins in this context can reveal relationships that may not otherwise be evident.

**Methods:**

We examined the human protein interaction network as it relates to human illness using the Disease Ontology. After calculating several topological metrics, we trained an alternating decision tree (ADTree) classifier to identify disease-associated proteins. Using a bootstrapping method, we created a tree to highlight conserved characteristics shared by many of these proteins. Subsequently, we reviewed a set of non-disease-associated proteins that were misclassified by the algorithm with high confidence and searched for evidence of a disease relationship.

**Results:**

Our classifier was able to predict disease-related genes with 79% area under the receiver operating characteristic (ROC) curve (AUC), which indicates the tradeoff between sensitivity and specificity and is a good predictor of how a classifier will perform on future data sets. We found that a combination of several network characteristics including degree centrality, disease neighbor ratio, eccentricity, and neighborhood connectivity help to distinguish between disease- and non-disease-related proteins. Furthermore, the ADTree allowed us to understand which combinations of strongly predictive attributes contributed most to protein-disease classification. In our post-processing evaluation, we found several examples of potential novel disease-related proteins and corresponding literature evidence. In addition, we showed that first- and second-order neighbors in the PPI network could be used to identify likely disease associations.

**Conclusions:**

We analyzed the human protein interaction network and its relationship to disease and found that both the number of interactions with other proteins and the disease relationship of neighboring proteins helped to determine whether a protein had a relationship to disease. Our classifier predicted many proteins with no annotated disease association to be disease-related, which indicated that these proteins have network characteristics that are similar to disease-related proteins and may therefore have disease associations not previously identified. By performing a post-processing step after the prediction, we were able to identify evidence in literature supporting this possibility. This method could provide a useful filter for experimentalists searching for new candidate protein targets for drug repositioning and could also be extended to include other network and data types in order to refine these predictions.

## Background

In the last several years, computational biology has made a variety of contributions to disease analysis using existing data in an attempt to increase our understanding of human illness. Popular topics include the identification and prediction of genes related to disease [1], statistical analysis of single nucleotide polymorphisms (SNPs) and disease [2], the prediction and discovery of new drug targets [3], the development of the disease ontology and its application to the human genome [4-6], the analysis of protein-protein interaction (PPI) networks as they relate to disease [7], and many others. The development of 'disease networks' [8,9], usually bipartite graphs describing disease as well as disease-gene relationships, have been of particular interest. In these networks, a connection between two diseases may signify one or more shared genes, proteins, metabolic pathways, microRNAs (miRNAs), or a number of other data types.

As opposed to many genetic disorders, complex disease types such as cancer and autoimmunity are often caused by the dysfunction of many biological systems at once. Proteins frequently cooperate in various ways to carry out DNA repair, gene regulation, epigenetic and histone modifications, metabolic pathways, and others vital cellular functions. Many complex diseases are related to each other via shared genes, meaning that the functional disruption of one gene product may result in multiple maladies. The disease outcome may also depend on a combination of protein dysfunctions. To confound the problem, not every gene is disease-causing when mutated and the exact character of a disease gene is still unclear. Due to the complicated nature of this problem, which is manually infeasible when examined on the proteomic level, researchers often employ machine learning methods to find solutions. If given descriptive characteristics of a set of instances, these algorithms can separate two classes of data, e.g., disease-related versus non-disease-related proteins. Several existing machine learning algorithms can help achieve this including support vector machines (SVM) [10], multiple instance learning [11], positive/unlabeled (PU) learning [12], Bayesian inference [13], and others. Ensemble classifiers can also be used to enhanced these methods, as we have done in previous work with the C4.5 decision trees [14], bootstrap aggregation [15] and cost-sensitive learning [16] where we predicted binding residues within DNA-binding proteins [17]. Recently, we found that the alternating decision tree algorithm, or ADTree, [18] worked well for analyzing methylation patterns on DNA [19], predicting a group of DNA-binding proteins [20], and identifying membrane-binding domains within protein families [21]. In each of these cases, this algorithm allowed us to identify the characteristics with the most influence on class determination for the examples by providing a graphical model of the decisions made by the classifier. A similar method can be useful in the case of disease-related gene identification.

There have been several previous attempts at global gene- and protein-disease association and prediction. Examples include the work of Özgür, Vu, Erkan, and Radev, who extracted disease genes from OMIM [22], overlaid the PPI network, and then used an SVM classifier with four centrality measures as features (degree, eigenvector, betweenness and closeness) to predict unknown disease genes [23]. Radivojac et al. used a protein interaction network with sequence and function data to infer disease-gene association [24]. Similarly, Furney et al. used knowledge of protein sequence and function to prioritize candidate cancer-related genes [25]. Gonzalez et al. predicted atherosclerosis-related genes based on connectivity by creating a protein interaction network and adding weights to certain proteins based on text mining of PubMed abstracts [26]. Xu and Li developed a K-nearest neighbor (KNN) classifier to predict hereditary disease genes from OMIM over the human PPI network with an overall accuracy of 76%. They found that these hereditary disease proteins tended to have a larger number of interactions and more shared neighbors than non-disease proteins [27]. Wu, Jiang, Zhang, and Li acquired disease-related genes from OMIM [22], identified these in the PPI network using HPRD [28], and then used linear regression and a concordance score to measure functional relatedness and phenotypic similarities between genes. In addition, they created CIPHER, a software tool that prioritizes disease genes [29]. Additional work in gene prioritization has employed random walk [30] and diffusion-based methods [31].

In this work we analyzed the currently known human protein interaction network and its relationship to disease using the ADTree algorithm. We used topological properties of this network to classify known disease-related proteins vs. non-disease related proteins. We then identified conserved rules over multiple trees to find the most discriminating characteristics of disease-related proteins in a network context. As a post-processing step, we examined the false positive examples that were assigned a high confidence score by the classifier and found that many of these proteins had potential disease associations. We then identified the most common diseases related to the first- and second-order neighbors of these proteins to further emphasize possible disease association.

## Methods

Toward the ultimate goal of identifying new potential disease-related genes, we developed a multi-stage protocol. First, we created a protein interaction network and calculated a set of topological properties for each protein to be used as predictive features. Second, we overlaid a set of known disease-related genes onto the network, identifying each protein product as 'disease-related' or 'non-disease related'. Third, we performed a binary classification on the proteins in the network using our feature set. During this process, each protein was predicted to belong to one of the two classes. Next, we ran a 10-fold cross validation over the model and used a bootstrapping technique to create a tree that highlights conserved rules. Subsequently, we evaluated the results from our classifier and ranked by confidence score those members of the negative class (non-disease-related proteins) that were misclassified as disease-related by our classifier. The reason for the misclassification is that the classifier recognized these proteins as having very similar characteristics to disease-related proteins based on the attributes provided. Therefore, a set of high-confidence false-positives can be considered potential novel disease-related proteins. We identified the first- and second-order neighbors for each of the high-confidence false positives. Next, we analyzed the distribution of diseases associated with these neighbors. Finally, we searched for evidence in literature that these misclassified proteins may play a role in some disease process.

### Data sets

We analyzed protein-protein interactions using the Human Protein Reference Database (HPRD) [28] Release 9, which contained 9,616 proteins and 39,240 binary interactions. We obtained disease-gene associations from DOLite [32]. These associations are based on a combination of the Disease Ontology [4] and the GeneRIFs (Gene Reference Into Function) construct (http://www.ncbi.nlm.nih.gov/gene/about-generif), which provides a short description of gene function and requires a published manuscript as supporting evidence. This combination, referred to as DORIF, was recently used to annotate the human genome [6]. They found that DORIF annotation provided a much higher recall rate when compared to OMIM data for validation gene sets. The DORIF annotation included 88,343 entries for 5,376 genes. There were 1,854 diseases and 48,436 PubMed references for gene-disease relationships. We created two groups for binary classification: those proteins with at least one assigned disease association (positive class) and proteins with no annotated disease association (negative class). 3,104 of these genes corresponded to a protein product in the PPI network, resulting in 32% of HPRD proteins having a disease association. Within this positive class the average number of diseases associations was 4.3 per protein. The average number of neighbors for the entire network was 7.7, the diameter of the network was 14, and the characteristic path length was 4.2. We created five versions of the data set, each with a different minimum number of disease associations required for inclusion in the positive class (see Additional file 1, Table S1). We analyzed the protein-protein interaction network using Cytoscape [33] and calculated nine features using Network Analyzer (http://med.bioinf.mpi-inf.mpg.de/netanalyzer/index.php) including degree, closeness, stress, and betweenness centralities, neighborhood connectivity, eccentricity, radiality, topological coefficient, and clustering coefficient. These are common methods for characterizing the importance, the influence, and the connectivity of as well as the distance between molecules in biological networks. These metrics are described in more detail in the supplementary material. We included an additional metric to describe the local environment of a node in terms of its disease-related neighbors. This feature, termed the disease neighbor ratio (DNR), was calculated as follows:

(1) $$DNR_{i}=\frac{n_{disease}}{\sum_{j=1}^{n}A_{ij}},$$

where *n_disease _*is the number of neighbors of node *i *identified as disease-related proteins, *n *is the number of nodes in the network, and *A *represents an adjacency matrix with elements *i *and *j*. The denominator is equivalent to the degree centrality of *i*.

Some metrics were excluded because they were very similar to ones we had chosen (for example, the all-pairs shortest path length [34]). Many other network statistics are available including eigenvector centrality and its variants [35-37] as well as composite [38] and integrative measures [39]. While arguments could be made for using different feature combinations depending on the goal of the experiment, we chose descriptive statistics that captured a variety of global and local characteristics of the protein interaction network. We made sure to include many of the most commonly used metrics in biological literature.

As a preliminary step, we identified the most distinguishing features in our data set using an attribute subset evaluator with a greedy step-wise search method within the Weka machine learning workbench [40]. The resulting attribute set in order of selection was disease neighbor ratio, degree, neighborhood connectivity, stress, topological coefficient, betweenness centrality, radiality, eccentricity, closeness centrality, and clustering coefficient.

### Machine learning: the alternating decision tree (ADTree)

**Figure 1:**
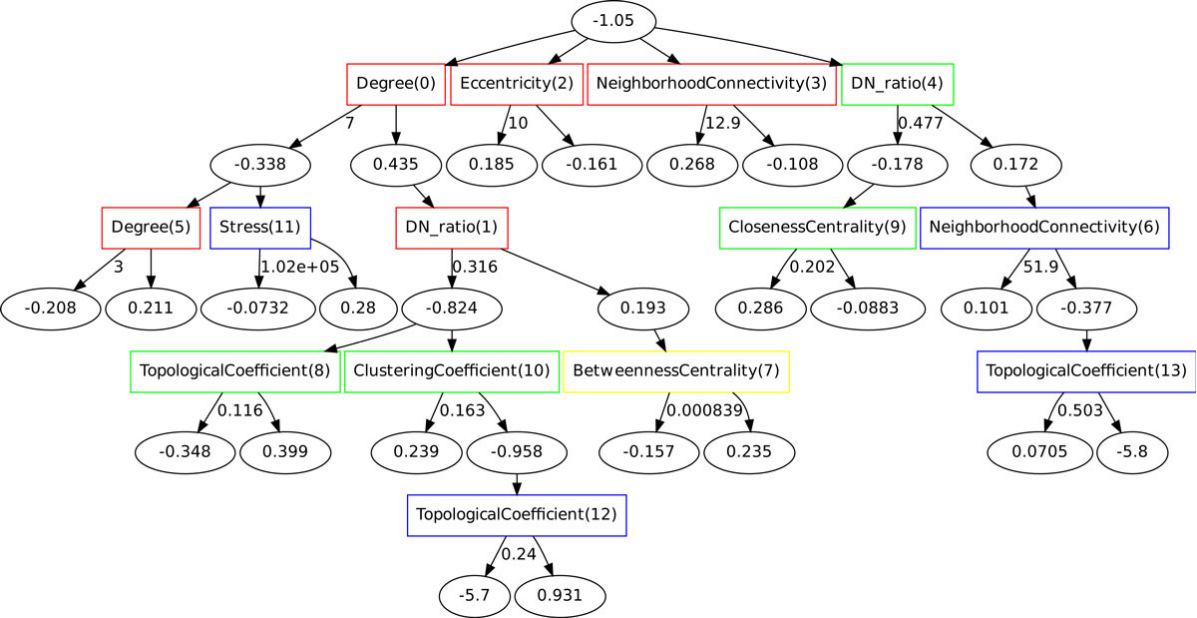
****An ADTree created using 10-fold CV and bootstrapping****. The root node indicates the bias in the data set, i.e., the ratio of positive to negative class examples (disease-associated proteins versus non-disease-associated proteins). The rectangles (decision nodes) contain the feature name. The number in parentheses within each decision node indicates the order in which the rule was found. The amount of node conservation between each of the trees generated in the validation step is indicated by the color of the box (red: ≥ 90%, orange: ≥ 70% (none in this tree), yellow: ≥ 50%, green: ≥ 30%, blue: ≥ 10%, black: ≤ 10% (none in this tree)). Ovals (prediction nodes) contain the value for the weighted vote, where a positive number indicates a prediction for disease-association. The numbers next to the arrows correspond to the threshold for the prediction. If the attribute value is equal to or exceeds this number, the left path is followed; otherwise the prediction follows the right path.

The ADTree [18] provides the benefits of a decision tree algorithm with the added advantage of an intuitive graphical model. This algorithm builds decision trees over a user-defined number of iterations using confidence-rated boosting, which results in an option tree [41]. The developed classifier returns both a class label and a score that measures the confidence in the classification. This confidence score is a sum of all scores acquired by the instance as it is evaluated using the rules in the tree. This additive score sets the ADTree apart from other decision trees in that instance classification is based on the entire path through the tree instead of one particular section of the path. A given instance is placed in the positive class if its final score is greater than 0, otherwise it is predicted to belong to the negative class (see Figure 1). We used the ADTree algorithm included in the MALIBU machine learning workbench [42]. We found that twenty tree-building iterations provided the best results with accuracy as the parameter selection standard. 10-fold cross validation was used for both the parameter selection and validation steps. Finally, we used a bootstrap sampling method to find conserved rules among multiple trees. These conserved rules corresponded to the most important features in determining the class of each instance.

### Classifier evaluation

We used a Receiver Operating Characteristic (ROC) curve to evaluate the performance of our models. The ROC curve measures the ability of a classifier to separate positive from negative examples and is generally considered a good measure of overall performance. The curve consists of continuous-valued outputs (corresponding to the likelihood for an example to belong to the positive class) from the generated model. The graph is formed by plotting the false positive rate (FPR, which is equal to 1 - specificity (Eq. 2)) versus the true positive rate (TPR, Eq. 3) for each example in the data set.

(2) $$ROC\;plot,\;X\;axis:\;FPR=\frac{FP}{FP+TN}$$

(3) $$ROC\;plot,\;Y\;axis:\;\;TPR=\frac{TP}{TP+FN}$$

An area under the ROC curve (AUC) of 0.5 is considered random, while an AUC equal to 1 would be characteristic of a flawless model. The AUC gives an idea of the tradeoff between sensitivity and specificity and is a good predictor of how a classifier will perform on future data sets.

## Results

### Disease-related protein classification

We created five versions of the disease-protein data set, each with an increasing number of disease associations required for a protein to belong to the group of positive examples (see Additional file 1, Table S1). We generated five classifiers using these data sets and performed 10-fold cross validation over each. Model performance increased with the removal of proteins associated with few diseases, which affected only the positive class in the prediction. After analyzing the ROC curves, we found that the classifier created using proteins associated with five or more diseases yielded the highest AUC but also had the widest ratio between positive and negative examples. The AUC for the five data sets were as follows: 67% for ≥ one disease, 71% for ≥ two diseases, 75% for ≥ three diseases, 76% for ≥ four diseases, and 79% for ≥ five diseases (see Additional file 1, Figure S1).

Next, we created a bootstrapped ADTree for the PPI-disease data (Figure 1). As indicated by the order in which the rules were found and by the conservation of rules discovered during the bootstrapping process, the attributes that were the most effective for distinguishing disease- from non-disease proteins were degree, disease neighbor ratio, eccentricity, and neighborhood connectivity. The next most conserved feature, present in at least 50% of the trees, was betweenness centrality. This feature was conserved when used in conjunction with degree and disease neighbor ratio. The remaining rules were conserved in ≤ 50% of the bootstrapped trees. Similar to other recent analysis [43,44], we found that the degree, disease neighbor ratio, and neighborhood connectivity metrics played an important role in the classification. In order to test the stability of the discovered rules, we removed 15% of the data set and reran the analysis. The results indicate that the rules within the trees remained largely consistent. The bootstrap method also helped to prevent large fluctuations between the final trees.

The bootstrapping process revealed rules that were conserved across the trees. These rules can give insight into potentially distinguishing characteristics of disease-related proteins. Two of the highly conserved rules involved only one attribute each: 'Eccentricity (2)' and 'Neighborhood Connectivity (3)'. Proteins that followed the first rule were identified as disease-related if they had an eccentricity value ≥ 10; otherwise they were identified as non-disease-related. Eccentricity measures the distance from the subject protein to the protein farthest away from it in the network. A high eccentricity value indicates that a protein is more isolated from others in the context of the network. Similarly, the classifier identified some proteins with a neighborhood connectivity score of 12.9 or greater as disease-related. This statistic measures the average number of proteins that interact with all neighbors of the subject protein. This rule suggests that some proteins may be disease-related because they are located within highly-connected subnetworks referred to as 'cliques' [45]. Other rules in the tree are more complicated and involve multiple interdependent attributes. One example is the rule involving 'Degree (0)', 'DN_ratio(1)', and 'Betweenness Centrality(7)'. For this rule, the proteins identified as disease-related with the highest confidence were those with six neighbors or fewer (less than 31% of which were in the disease-related class) that did not tend to bridge subnetworks to each other in the network. Though the degree attribute itself was a strong predictor, the confidence score was increased when the additional two criteria were met.

The majority of the rules in the tree suggest that disease genes tend not to be highly connected to other genes in the network but rather lie near the perimeter and are therefore less likely to be vital to the structure of the network. This is in agreement with previous analysis of the human disease-gene network using the OMIM database [8]. However, in our data set we found that, overall, disease-related proteins tended to have a higher degree (i.e., more interactions with other proteins) and disease neighbor ratio compared to non-disease proteins (see Additional file 1, Figure S2). This difference could be due not only to a few highly connected proteins but to the fact that in contrast to the OMIM data set, which includes only genetic disorders, our data set includes both genetic and complex diseases, which can involve many genes.

To test the importance of the most discerning features, we ran the algorithm four more times, each time removing one of these important attributes. Removing the disease neighbor ratio resulted in an 11% decrease in sensitivity (which measures the ratio of true positive examples and those correctly identified as positive). Removal of the degree centrality and neighborhood connectivity features reduced sensitivity by 3% each. These results along with the ADTree in Figure 1 make it clear that while individual attributes may contribute more or less to a prediction problem, the combination of these features gives us a multi-dimensional view of how the two classes are separated.

### Comparison with other algorithms and previous results

**Figure 2:**
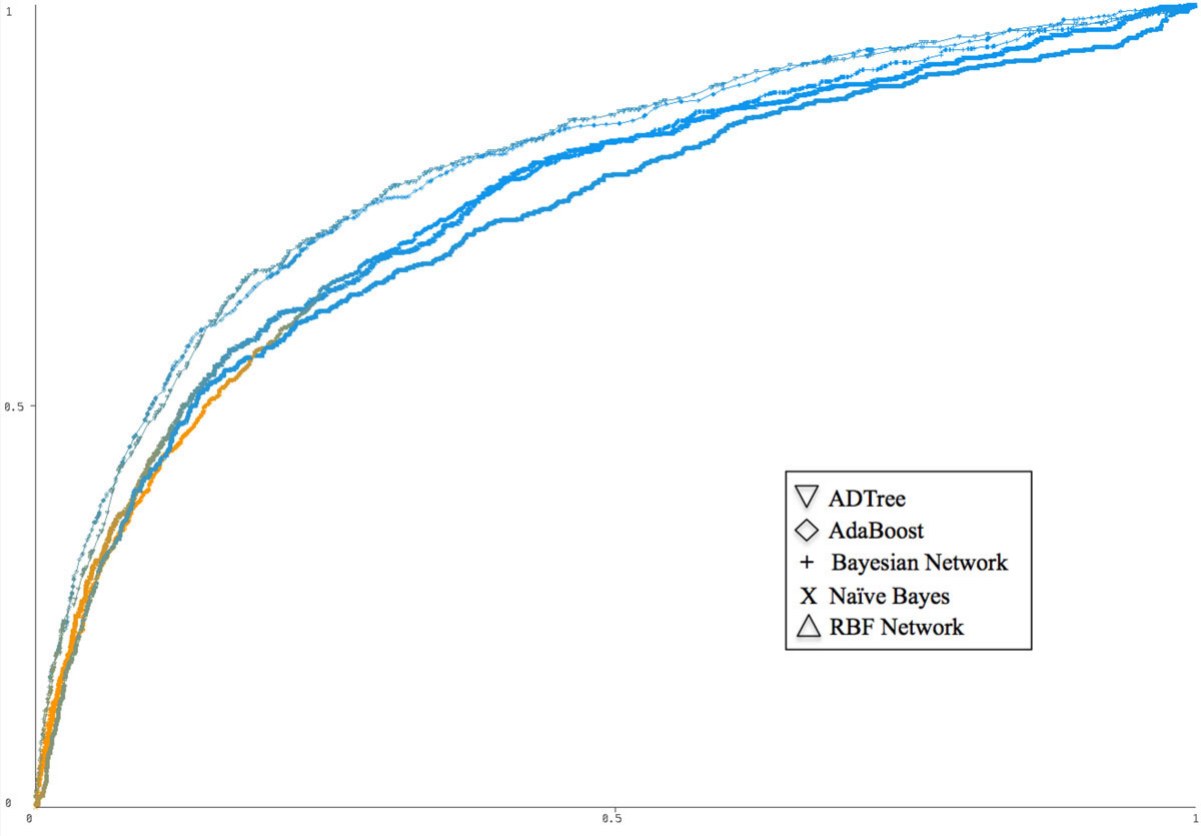
**ROC curves comparing five classifiers run over the disease-protein network data set**. The top two performers were ADTree and AdaBoost (both AUC = 0.795), followed by the Bayesian network and the Naïve Bayesian classifiers (both AUC = 0.754), and finally the RBF network (AUC = 0.726). The curves are colored according to the threshold value and based on a color gradient scale from blue (threshold value of 0) to orange (threshold value of 1). This figure was created using Weka [40].

Due to both the wide variety of methods used in gene-disease association studies and the high variability of the data sets used for evaluation, direct comparison with the results of previous work was not feasible. Instead, we compared the performance of the ADTree algorithm on our data set with that of a variety of other tree-based classifiers as well as Bayesian, function-based, and meta-classifiers using the Weka machine learning workbench [40]. Figure 2 shows ROC curves for the ADTree, Adaboost [46], Bayesian Network, Naïve Bayes, and Radial Basis Function (RBF) Network [47] classifiers. ADTree and AdaBoost performed similarly (AUC = 0.795), as did the Bayesian Network and Naïve Bayes methods (AUC = 0.754). The poorest performance in the group was that of the RBF Network (AUC = 0.726). While AdaBoost was capable of producing the same area under the ROC curve as ADTree, the ADTree provides the benefit of an interpretable model that describes the interdependency of features.

In addition to comparisons with other algorithms, we examined the results of our classifier and found that we correctly identified 17/17 disease-related proteins that Gonzalez et al. [26] mined from literature. Our classifier also correctly predicted 14/16 known breast cancer genes identified by Wu et al. [29]. Additionally, we were able to correctly classify 15/16 known disease genes found in literature by Özgür et al. [23].

### Identification of potential disease genes

Our classifier predicted 98 non-disease-related proteins (i.e., those that lack DORIF annotation) to be members of the positive class with a confidence score ≥ 0.5 (threshold = 0). This indicated that these examples have attribute values that qualify them as potential disease-related proteins. We examined the fifteen false positive examples with the highest confidence score more carefully using the MalaCards [48] and GeneCards [49] databases and found that there was evidence linking many of these proteins to disease. Table 1 shows this group ranked by confidence. Only two proteins (PTCH1 and TCF4) have associated MIM numbers (indicating Mendelian disease involvement). Nine out of fifteen proteins (CDH5, DPP4, GZMB, FGR, FLT1, PECAM1, SREBF2, STAT6, and TOP1) have moderate to strong evidence of disease association, while four of the fifteen (STAMBPL1, MDH2, GRK5, and CD74) have light evidence. Interestingly, twelve of these proteins are linked to some form of cancer or tumor development.

**Table 1:** A subset of negative-class proteins predicted to be disease-related

Conf. Score	OS	DORIF	OMIM	Suspected Disease Relationship
6.24096	CDH5	-	-	Melanoma, tumor metastasis
5.8186	PTCH1	-	109400, 605462, 610828	Basal Cell Nevus Syndrome, Basal Cell Carcinoma
5.19721	STAMBPL1	-	-	Very light evidence, Alzheimer's
5.14813	MDH2	-	-	Very light evidence, tumor development
1.09972	DPP4	-	-	Diabetes (17 PMIDs), colon cancer (3 PMIDs)
1.09972	GRK5	-	-	Very light evidence, heart failure
0.907016	GZMB	-	-	Lymphoma (30 PMIDs), tumors (92 PMIDs)
0.898631	TCF4	-	610954	Pitt-Hopkins Syndrome, various cancer (light evidence)
0.705929	FGR	-	-	Breast cancer (3 PMIDs), prostate cancer (1 PMID)
0.705929	FLT1	-	-	Cancer, various
0.705929	PECAM1	-	-	Cancer, various
0.705929	SREBF2	-	-	Prostate cancer (2 PMIDs)
0.705929	STAT6	-	-	Prostate cancer (3 PMIDs)
0.705929	TOP1	-	-	Leukemia, colon and ovarian cancer
0.664823	CD74	-	-	Very light evidence, lymphoma

A subset of 15 proteins belonging to the non-disease-related class (lacking DORIF annotation) but predicted to be disease-related, sorted by the ADTree-assigned confidence score. Two proteins (PTCH1 and TCF4) have associated OMIM disorders. 9/15 proteins (CDH5, DPP4, GZMB, FGR, FLT1, PECAM1, SREBF2, STAT6, and TOP1) have moderate to strong evidence of disease association, while 4/15 (STAMBPL1, MDH2, GRK5, and CD74) have light evidence linking them to disease. (*n *PMIDs) indicates the number of PubMed IDs connecting a protein to a particular disease. 'Conf. Score' is the confidence score assigned by the ADTree classifier, 'OS' is the official symbol of the gene, 'DORIF' is Disease Ontology + Gene Reference Into Function, 'OMIM' is the MIM number associated with the gene, 'light evidence' is defined as having a predicted disease association according to the MalaCards database [48]. Disease information for this table was acquired from the GeneCards database [49].

**Figure 3:**
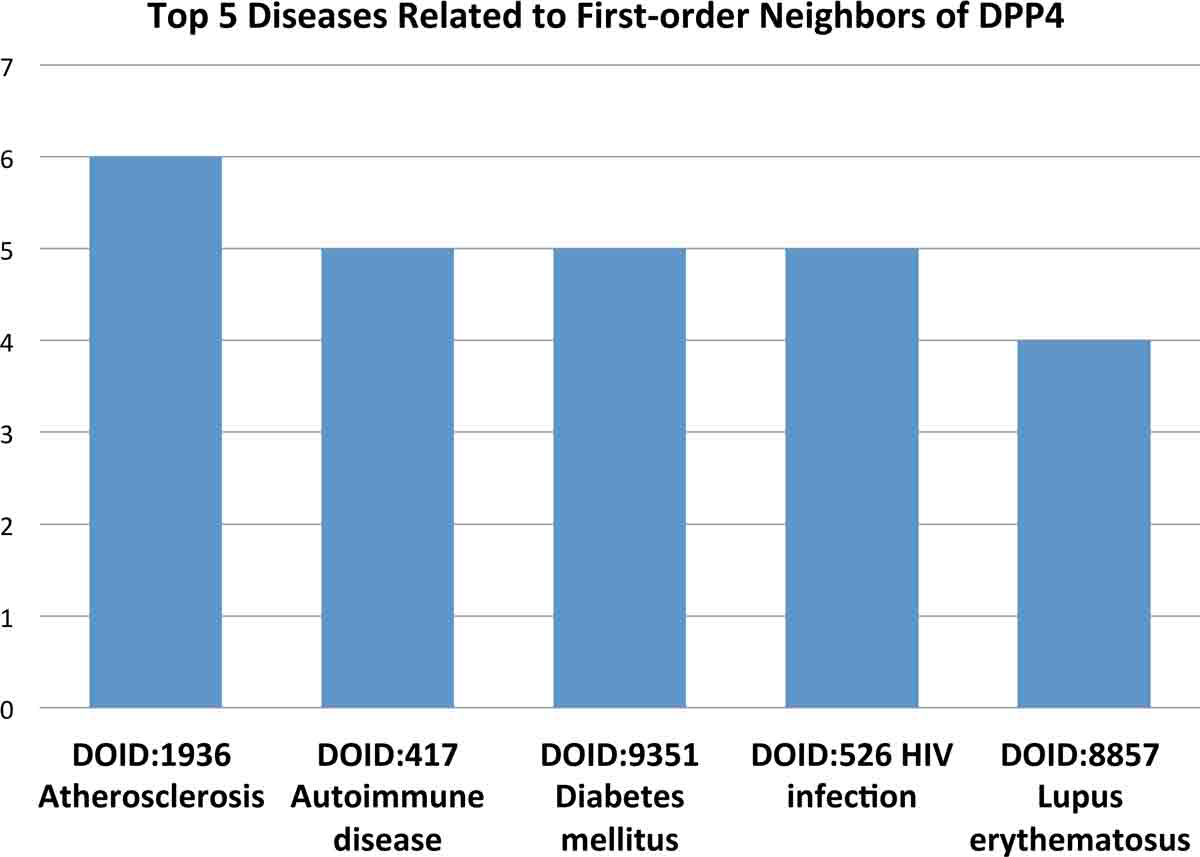
**The five most common diseases associated with the first-order neighbors of DPP4**. The five most common diseases associated with the first-order neighbors of DPP4 (those proteins with a direct interaction). DPP4 has 55 PubMed IDs that associate it with non-insulin-dependent diabetes mellitus (NIDDM). Interestingly, NIDDM is often accompanied by beta cell autoimmunity, where the beta cells of the pancreas are destroyed by an autoimmune disorder [50].

**Figure 4:**
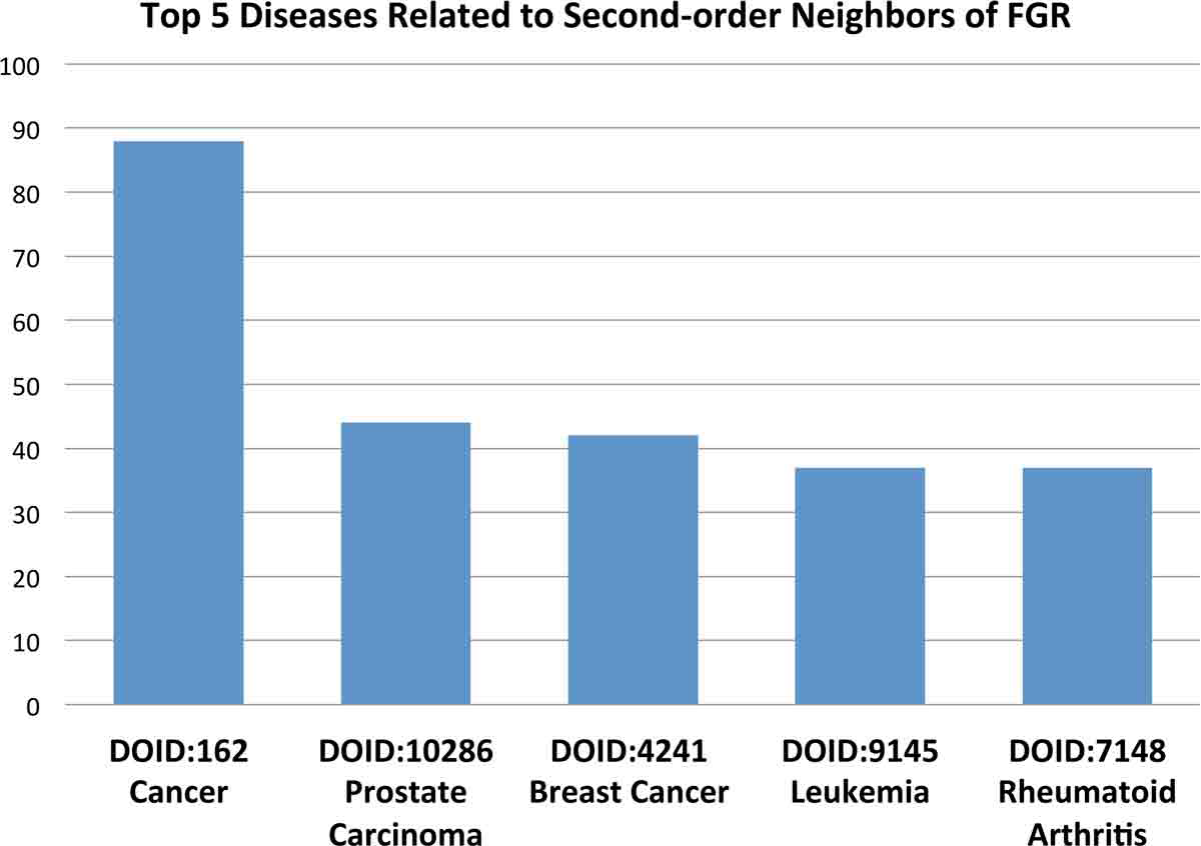
**The five most common diseases associated with the second-order neighbors of FGR**. The five most common diseases associated with the second-order neighbors (i.e., neighbors of neighbors) of FGR. There are three PMIDs associating this gene with breast cancer and one PMID linking it to prostate cancer.

Other proteins within the network neighborhood can offer clues about potential disease associations of these misclassified proteins. For example, we identified the first-order neighbors (i.e., proteins with a direct interaction) of dipeptidyl-peptidase 4 (DPP4, Figure 3). This gene product is a glycoprotein receptor involved in the signaling pathway for T-cell receptor (TCR)-mediated T-cell activation [49]. DPP4 has 55 PubMed IDs associating it with non-insulin-dependent diabetes mellitus (NIDDM). Figure 3 shows the five most common diseases of DPP4 first-order neighbors by Disease Ontology ID (DOID). 'Diabetes mellitus' ranks third, while 'Autoimmune disease' ranks second. Interestingly, NIDDM is often accompanied by beta cell autoimmunity, where the beta cells of the pancreas are destroyed by an autoimmune disorder [50]. We used a similar method for the Gardner-Rasheed feline sarcoma viral (v-fgr) oncogene homolog (FGR), but this time we examined the second-order neighbors of the protein (i.e., neighbors of neighbors). There are three PubMed IDs (PMIDs) associating this gene with breast cancer and one PMID linking it to prostate cancer. Figure 4 shows the top five diseases related to the second-order neighbors of FGR. 'Prostate Carcinoma' and 'Breast Cancer' are the second and third most common diseases, respectively, only behind the general category of 'Cancer'.

**Figure 5:**
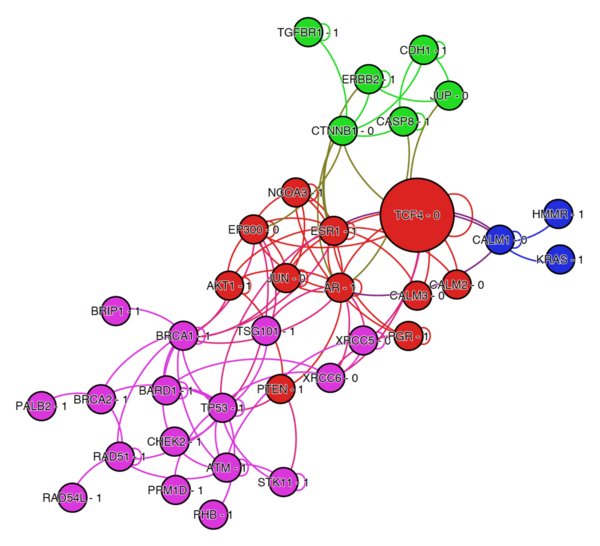
**The network neighborhood of the transcription factor TCF4**. A subset of proteins from the PPI-disease data set highlighting the relationship between breast cancer-related genes and the transcription factor TCF4, one of 15 proteins in our set of high-confidence false positive predictions (Table 1). Proteins are colored according to modularity class (four modules were identified). Proteins are labeled with the gene's official symbol with a '- 1' afterwards to indicate breast cancer association and a '- 0' to indicate no association. The TCF4 node has been made larger for identification purposes.

For further analysis, we examined the transcription factor TCF4, which also belonged to our high-confidence set of false positives (Table 1). The TCF4 gene, which encodes a protein known as transcription factor 4, is one of only two genes in our set to have an MIM number assigned and has been implicated in Pitt-Hopkins Syndrome, a condition that results in severe intellectual and physical disabilities [51]. When analyzed in the context of the PPI network, we observed that TCF4 had a high number of second-order neighbor proteins related to breast cancer. We extracted a subnetwork of proteins which included those identified as breast cancer-related by OMIM (MIM:114480), those identified as such in the work of Wu et al. [29], and genes tested for breast cancer-related mutations at My Cancer Genome (http://www.mycancergenome.org), a personalized cancer medicine resource managed by the Vanderbilt-Ingram Cancer Center. In addition, we included a set of eleven first-order neighbor proteins for TGF4. The resulting network (Figure 5), visualized using the network software Gephi [52], consisted of 36 nodes and 107 edges. We used a community detection algorithm [53] to partition the network into modules and colored the proteins by modularity class. Interestingly, TCF4 has only one first-order neighbor identified as breast cancer-related, the androgen receptor AR. A literature search revealed that TCF4 and AR have been shown to interact via the DNA-binding domain of AR [54]. AR is known to be expressed in many breast tumors [55] and is seen as a potential drug target [56]. There is also evidence that TCF4 may have a role in breast cancer progression due to its interaction with the β-catenin protein (encoded by the CTNNB1 gene) and the Wnt signaling pathway [57]. This example illustrates how an initial classification step can help to direct a network neighborhood search that facilitates protein-disease association discovery.

## Discussion

The benefits of the ADTree algorithm are two-fold: first, it provides a confidence score for each example based on its full traversal path through the tree. Second, it allows us to identify interdependencies between attributes in the prediction and illustrates a pathway by which rules work together to discriminate between disease- and non-disease-related proteins. We found that, due to the difficult nature of this prediction problem and complexity of the data set, it was necessary to add a post-classification processing step to evaluate false positive predictions. Interestingly, the confidence score helped to point out non-disease-related proteins that may in fact be disease-related. The examples provided by DPP4, FGR, and TCF4 illustrate how the PPI network can be used during this post-processing step to examine the network neighborhood of potential disease-related proteins and to identify disease(s) with which these proteins may be associated.

An obvious weakness of this approach is the effect of data set bias during classification, which is a result of the tendency for highly studied proteins to be overrepresented. Also, any change in the structure of the network will change its topological properties, and, because the initial identification of potential disease genes is based on these properties, network statistics should be recalculated following any addition or deletion of nodes or edges. Also, it is important to note that a 'disease-related protein' is not necessarily the cause of a particular disease. The role that a protein product plays in a disease process may depend on specific mutations to its corresponding gene or that of its interacting partners, tissue specificity, conditional essentiality, and other factors. The prediction of disease-related genes is a first step in a process that includes experimental evaluation. The advantage of the prediction step is that it acts as a filter and focuses effort toward those proteins that are more likely to play important roles in disease, as well as those proteins that may serve as potential drug targets.

As we have learned from our work, diseases share interactions through molecular networks. One of the next steps in disease-gene analysis could be to study connections between diseases; for instance, various types of cancers as they relate to other illnesses such as diabetes [58], various infections [59], and obesity [60,61]. Though the type or nature of this relationship may be unknown, we may be able to shed light on the subject using these knowledge-mining methods along with molecular data such as metabolic pathways, regulation networks, and others. We believe that as more complete data sets become available, a higher level of knowledge will be attainable by utilizing this method.

## Conclusions

We analyzed the human protein interaction network and its relationship to disease and found that both the number of interactions with other proteins and the disease relationship of neighboring proteins helped to determine whether a protein had a relationship to disease. Our classifier predicted many proteins with no annotated disease association to be disease-related, which indicated that these proteins have network characteristics that are similar to disease-related proteins and may therefore have disease associations not previously identified. By performing a post-processing step after the prediction, we were able to identify evidence in literature supporting this possibility. This method could provide a useful filter for experimentalists searching for new candidate protein targets for drug repositioning and could also be extended to include other network and data types in order to refine these predictions.

## List of abbreviations used

ADTree: Alternating decision tree

PPI: Protein-protein interaction

ROC: Receiver operating characteristic

AUC: Area under the ROC curve

## Competing interests

The authors declare that they have no competing interests.

## Authors' contributions

MBC prepared, wrote, and edited the manuscript, designed the experiments, and executed the experiments.

HL designed the experiments and edited the manuscript.

## Supplementary Material

Additional file 1 **Microsoft Word Document (**.docx), supplementary_material. This file provides further description of the features used in this work as well as two supplementary tables and two figures. Click here for file

## Declarations

Publication of this article has been funded by the National Natural Science Foundation of China (Grant No. 31071167).

This article has been published as part of *BMC Medical Genomics *Volume 8 Supplement 2, 2015: Selected articles from the 4th Translational Bioinformatics Conference and the 8th International Conference on Systems Biology (TBC/ISB 2014). The full contents of the supplement are available online at http://www.biomedcentral.com/bmcmedgenomics/supplements/8/S2.
