# Supplementary material for: Network-based prediction and knowledge mining of disease genes
Source: BMC Med Genomics. 2015 May 29;8(Suppl 2):S9. doi: 10.1186/1755-8794-8-S2-S9 (PMC4460923; doi:10.1186/1755-8794-8-S2-S9)
Supplement: Additional file 1 — Microsoft Word Document (.docx), supplementary_material. This file provides further description of the features used in this work as well as two supplementary tables and two figures. [file 1755-8794-8-S2-S9-S1.docx]

## SUPPLEMENTARY MATERIAL

## Features

We used ten topological features to build our ADTree model. The first nine were calculated using Network Analyzer (<http://med.bioinf.mpi-inf.mpg.de/netanalyzer/index.php>). The tenth feature, which we call the disease neighbor ratio (DNR), was calculated separately. Each is described below.

**Degree centrality**

The degree of a vertex is its total number of edges. In our network, which was undirected, edges represented molecular interactions between proteins. The degree *k* of vertex *i* can be described in this case as

$k_{i}=\sum_{j=1}^{n} A_{ij}$ (1)

where *n* is the number of nodes in the network and *A* represents an adjacency matrix with elements *i* and *j*. This measure has been shown in several works to be a distinguishing characteristic between disease and non-disease genes [1-4].

**Closeness centrality**

Closeness centrality describes the average distance between a given node and other nodes in a network, where 0 ≤ *x* ≤ 1. Because it is an inverse measure, a larger value indicates a lower level of centrality. It can be thought of as a measure of the rate at which information spreads to neighboring nodes [5]. Closeness can be defined as

$C_{c}\left( n \right)=\frac{1}{avg(L\left( n,m \right))}$ (2)

where *L(n,m)* indicates the shortest path distance between nodes *n* and *m*. This measure has been used by Ortutay and Vihinen [3] to identify primary immunodeficiency-related genes.

**Betweenness centrality**

This measure indicates the number of shortest paths through each vertex. Nodes with high betweenness centrality (often called bottlenecks) have been shown to correspond to essential genes in directed networks [1]. Betweenness centrality can be written as

$C_{b}\left( n \right)=\sum_{s\neq n\neq t} (\frac{\sigma_{st}(n)}{\sigma_{st}})$ (3)

where *s* and *t* are vertices other than *n*, *σ_st_* represents the shortest path count, and *σ_st_ (n)* is the shortest path count from *s* to *t* with which *n* is involved.

**Clustering coefficient**

The clustering coefficient (CC) of a node *n* is the ratio of the existing edges between n and its neighbors and the number of possible connections. This is a measure of edge density for a node neighborhood [2, 4, 6]. The clustering coefficient of *n* for an undirected network can be written as

${CC}_{n}=\frac{2e_{n}}{(k_{n}\left( k_{n}-1 \right))}$ (4)

where *k_n_* is the total neighbor count of node *n* and *e_n_* is the count of linked pairs of nodes between neighbors of *n* [7, 8].

**Stress centrality**

The stress centrality [9, 10] for a node *n* corresponds to the number of shortest paths traveling through it. If this number is large, the stress value will be large as well. This metric is described in the following way:

$C_{s}\left( n \right)=\sum_{s\neq n} \sum_{t\neq n,s} \sigma_{st}(n)$ (5)

where *s* and *t* are network vertices other than *n*, *σ_st_* indicates the shortest path count from vertex *s* to vertex *t*, and *σ_st_ (n)* is the shortest path count from *s* to *t* passing through *n*.

**Neighborhood connectivity**

The neighborhood connectivity of a vertex is equal to the average degree of its neighbors [11].

**Topological coefficient**

This metric describes the average number of shared connections between a node and other nodes. In a social context this would be equivalent to the number of mutual friends two people share. The topological coefficient [12] can be represented as

$T_{n}=\frac{avg(J\left( n,m \right))}{k_{n}}$ (6)

where *k_n_* indicates the neighbors of node *n* and *J(n,m)* is the total count of neighbors that *n* and *m* share. ‘1’ is added if *n* and *m* share an edge. *J(n,m)* is defined only for the group of nodes *m* that have at least one neighbor in common with *n*.

**Eccentricity**

Eccentricity is the longest path between a node *n* and another node. The eccentricity value is 0 for isolated nodes, while the maximum value is the diameter of the network. The eccentricity of a node *n* is defined as

$C_{ecc}\left( n \right)=\frac{1}{max\{dist\left( v,w \right) :w \in V}$ (7)

**Radiality**

Radiality is a measure of centrality [9, 13], where 0 ≤ *C_rad_* ≤ 1. It is calculated as the average shortest path length (ASPL) of a node *n* minus the connected component diameter plus one. A high radiality value indicates that a vertex can easily reach other vertices [14].

$C_{rad}\left( n \right)=\frac{\sum_{w\in V} (\in_{G}+1-dist\left( v,w \right))}{n-1}$ (8)

**Disease neighbor ratio**

We included this additional metric to describe the local environment of a node in terms of its disease-related neighbors. We represent the disease neighbor ratio (DNR) as

${DNR}_{i}=\frac{n_{disease}}{\sum_{j=1}^{n} A_{ij}}$ (9)

where *n_disease_* is the number of neighbors of node *i* identified as disease-related proteins, *n* is the number of nodes in the network, and *A* represents an adjacency matrix with elements *i* and *j*. The denominator is equivalent to the degree centrality of *i*.

**Supplementary Tables and Figures:**


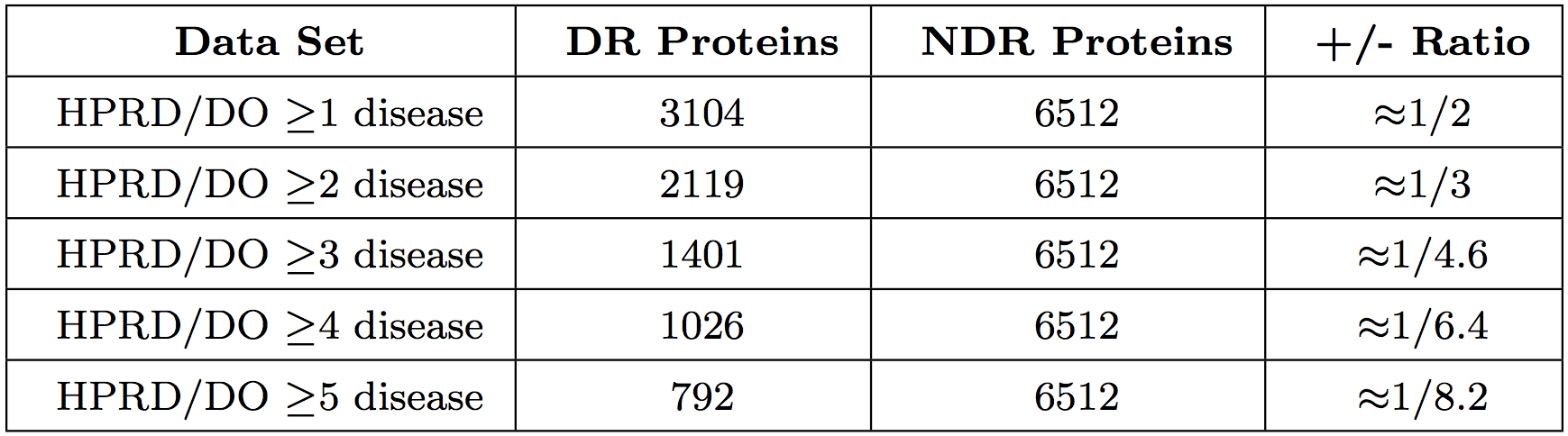


## Table S1 - Data set composition

We created five versions of the PPI data set. “≥ *n*” in the data set name indicates the number of diseases that must be associated with a protein for it to be a member of the positive class. “DR” indicates the number of disease-related proteins (+ class), “NDR” indicates the number of non-disease-related proteins (- class), and “+/- Ratio” indicates the ratio of positive and negative class examples for a data set.

| **Proteins with…** |  | **DNR** | **# of Disease Neighbors** | **Disease Count** |
| --- | --- | --- | --- | --- |
| **≥ 5 disease associations (+)** | **Mean** | 0.52704 | 9.3098 | 11.148 |
|  | **Median** | 0.52174 | 5 | 8 |
|  | **Mode** | 0.5 | 1 | 5 |
| **No disease association (-)** | **Mean** | 0.40899 | 2.6381 | 0 |
|  | **Median** | 0.36364 | 1 | 0 |
|  | **Mode** | 0 | 1 | 0 |

**Table S2 – Disease-related statistics for the two classes in data set 5**

Statistics for the disease neighbor ratio, the number of disease-associated neighbors, and the number of associated diseases for proteins associated with five or more diseases vs. those associated with no diseases.


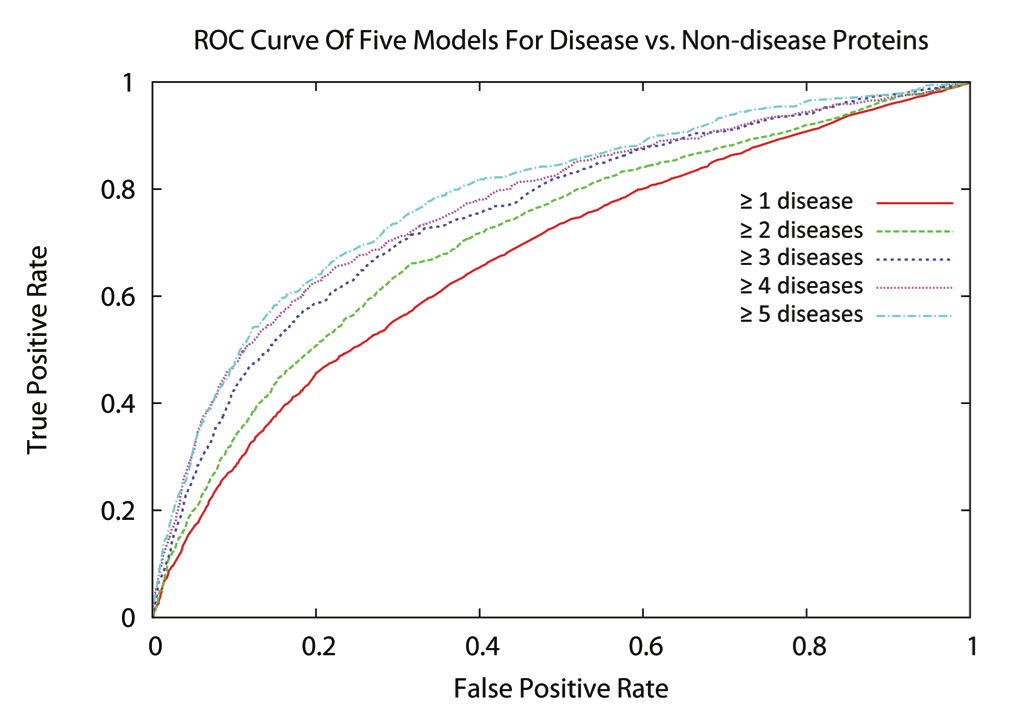


## Figure S1. ROC curves for five ADTree classifiers

We created five versions of the disease-protein data set, each with an increasing number of disease associations required for a protein to belong to the group of positive examples. We generated five classifiers using these data sets and performed 10-fold cross validation over each. Model performance increased with the removal of proteins associated with few diseases, which affected only the positive class in the prediction. After analyzing the ROC curves, we found that the classifier created using proteins associated with five or more diseases yielded the best results. The AUC for the five data sets were as follows: 67% for ≥ one disease, 71% for ≥ two diseases, 75% for ≥ three diseases, 76% for ≥ four diseases, and 79% for ≥ five diseases.


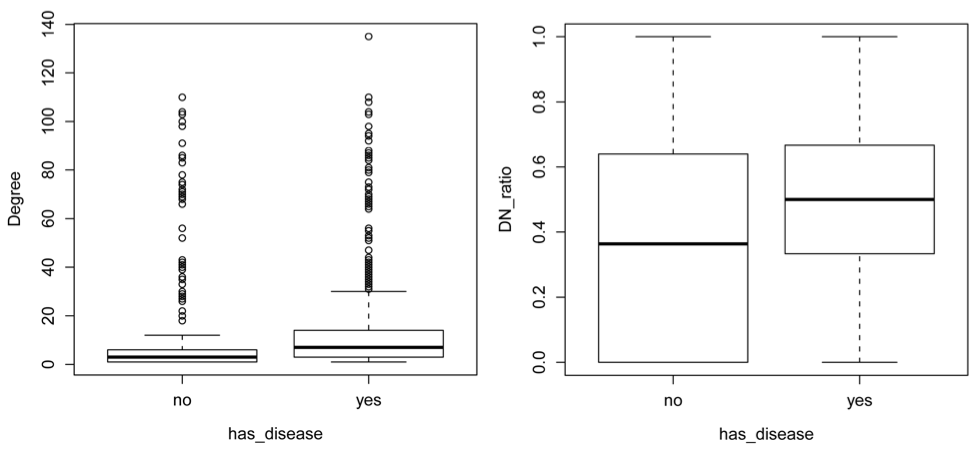


## Figure S2 - Box plots showing two of the most distinguishing features between disease and non-disease proteins

Box plots for degree centrality and disease neighbor ratio. These features correspond to the first and second rules found using the ADTree model, respectively (created using the R statistical environment [15]). Disease-related proteins had a higher degree centrality and disease neighbor ratio compared to non-disease proteins.

**SUPPLEMENTARY REFERENCES**

1. Barabasi AL, Gulbahce N, Loscalzo J: **Network medicine: a network-based approach to human disease.** *Nat Rev Genet* 2011, **12:**56-68.

2. Feldman I, Rzhetsky A, Vitkup D: **Network properties of genes harboring inherited disease mutations.** *Proc Natl Acad Sci U S A* 2008, **105:**4323-4328.

3. Ortutay C, Vihinen M: **Identification of candidate disease genes by integrating Gene Ontologies and protein-interaction networks: case study of primary immunodeficiencies.** *Nucleic Acids Res* 2009, **37:**622-628.

4. Li L, Zhang K, Lee J, Cordes S, Davis DP, Tang Z: **Discovering cancer genes by integrating network and functional properties.** *BMC Med Genomics* 2009, **2:**61.

5. Newman MEJ: *Networks: An Introduction.* New York, NY, USA: Oxford University Press, Inc.; 2010.

6. Ideker T, Sharan R: **Protein networks in disease.** *Genome Res* 2008, **18:**644-652.

7. Watts DJ, Strogatz SH: **Collective dynamics of 'small-world' networks.** *Nature* 1998, **393:**440-442.

8. Barabasi AL, Oltvai ZN: **Network biology: understanding the cell's functional organization.** *Nat Rev Genet* 2004, **5:**101-113.

9. Brandes U: **A Faster Algorithm for Betweenness Centrality.** *Journal of Mathematical Sociology* 2001, **25:**163-177.

10. Shimbel A: **Structural parameters of communication networks.** *Bulletin of Mathematical Biology* 1953, **15:**501-507.

11. Maslov S, Sneppen K: **Specificity and stability in topology of protein networks.** *Science* 2002, **296:**910-913.

12. Stelzl U, Worm U, Lalowski M, Haenig C, Brembeck FH, Goehler H, Stroedicke M, Zenkner M, Schoenherr A, Koeppen S, et al: **A human protein-protein interaction network: a resource for annotating the proteome.** *Cell* 2005, **122:**957-968.

13. Valente TW, Foreman RK: **Integration and radiality: Measuring the extent of an individual's connectedness and reachability in a network.** *Social Networks* 1998, **20:**89-105.

14. Koschutzki D, Schreiber F: **Centrality analysis methods for biological networks and their application to gene regulatory networks.** *Gene Regul Syst Bio* 2008, **2:**193-201.

15. RCoreTeam: **R: A Language and Environment for Statistical Computing.** In *Book R: A Language and Environment for Statistical Computing* (Editor ed.^eds.). City: R Foundation for Statistical Computing; 2012.
